# Lipidomic Profiling of Di- and Tri-Acylglycerol Species in Weight-Controlled Mice

**DOI:** 10.1371/journal.pone.0116398

**Published:** 2015-02-23

**Authors:** Brenee S. King, Lizhi Lu, Miao Yu, Yu Jiang, Joseph Standard, Xiaoyu Su, Zhihui Zhao, Weiqun Wang

**Affiliations:** 1 Department of Human Nutrition, Kansas State University, Manhattan, KS 66506, United States of America; 2 Institute of Animal Husbandry & Veterinary Science, Zhejiang Academy of Agricultural Sciences, Hangzhou 310021, China; 3 Institute for Agri-food Standards and Testing Technology, Shanghai Academy of Agricultural Sciences, Shanghai 201403, China; Universidad Pablo de Olavide, Centro Andaluz de Biología del Desarrollo-CSIC, SPAIN

## Abstract

Weight control by dietary calorie restriction (DCR) or exercise has been shown to prevent cancer in various models. However, the mechanisms as to how weight control is beneficial are not well understood. While previous reports have investigated the effects of weight control on total lipid levels or lipid composition within cellular membranes, there has been little work surrounding changes to individual lipids following weight control interventions. In this study, using a model of skin carcinogenesis centered on the tumor promotion stage, CD-1 mice were randomly assigned into 4 groups: ad libitum and sedentary (control), ad libitum with exercise (AL+Exe), exercise with pair feeding of a diet isocaloric with control (PF+Exe), and sedentary with 20% DCR compared to control. After ten weeks, body weight and body fat percentages significantly decreased in the PF+Exe and DCR groups but not AL+Exe when compared with sedentary controls. Murine skin and plasma samples were obtained for analysis. Lipidomics using electrospray ionization MS/MS was employed to profile triacylglycerol (TG) and diacylglycerol (DG) species. Both plasma and tissue TG species containing fatty acid chains with length 18:1 were significantly decreased following DCR when compared to sedentary control animals. In regards to DG, the most significant changes occurred in the plasma. DG species containing fatty acids with lengths 16:1 or 18:1 were significantly decreased in PF+Exe and DCR groups when compared to sedentary controls. Due to the significant role of TG in energy storage and DG in cellular signaling, our findings of the effects of weight control on individual TG and DG species in plasma and skin tissue following exposure to a tumor promoter, may provide insight into the mechanism of weight control on cancer prevention.

## Introduction

Body weight is influenced by energy balance, which is tightly associated with energy intake, energy expenditure, and energy digestibility. Common ways to modify body weight include exercise (energy expenditure) and/or a changes to one’s eating (energy intake). There are several advantages to body weight control, one being a possible decrease in the risk of various cancers including colon, endometrium, breast (postmenopausal), esophagus, and kidney [1]. Studies investigating the effects of exercise/physical activity on cancer prevention have been conducted using animal models and humans [2–3]. Dietary calorie restriction (DCR) which involves reducing one’s intake of calories has recently (past 30+ years) been shown to be a potent suppressor of carcinogenesis [4–5]. The molecular mechanisms associated with the beneficial effects of exercise and/or DCR on cancer prevention are continually being investigated. Several suggested mechanisms include reductions to fat storage, increases in immune defense, reduction in inflammatory pathways and modifications to growth factor signaling pathways such as IGF-1 [3, 5].

Using a mouse model of skin carcinogenesis promoted by 12-O-tetradecanoyl-13-phorbol acetate (TPA), previous work in our lab, and others, show the cancer preventative effects of DCR and exercise can occur during the tumor promotion stage of carcinogenesis. More specifically, our data show there are modifications to the IGF-1 signaling pathway which can subsequently activate the phosphatidylinositol 3-kinase (PI3K)-AKT pathway, resulting in phosphorylation of cell membrane phosphoinositides (PI) for cell cycle progression and cell survival [6–7]. While signaling of phospholipids is known to be important in cancer progression, studies have also investigated the role of fatty acid lipid composition within cellular membranes and signaling processes. Central to both these processes are triacylglycerols (TG) and diacylglycerols (DG).

Triacylglycerols (TG) are composed of a glycerol molecule linked to three fatty acids through ester bonds. The major roles of TG include storage of energy, storage of fatty acids, and provision of precursors for phospholipid biosynthesis. Critical enzymes involved in fatty acid and TG biosynthesis are regulated by hormonal, developmental and nutritional conditions [8–9]. Decreases in plasma TG have been observed in weight control via calorie restriction. Thirty percent calorie restriction decreased plasma TG and fatty acid concentrations, as well as triacylglycerol accumulation in the liver [10–11]. Additionally, exercise has been shown to affect plasma TG levels [12]. Haskell et al. showed that exercise may increase lipoprotein lipase activity in skeletal muscle and adipose tissue, and decrease hepatic TG synthesis [13].

Diacylglycerols (DG) consist of an ester derived from two long-chain fatty acids. DG are minor components in most tissues; however, they are important intermediates in lipid metabolism and key elements in cell signaling [14]. Within the cell membrane, DG can modify bilayer properties by facilitating membrane fusion/fission and affect intracellular vesicular trafficking [14–15]. DG has been studied extensively as a lipid second massager. Following stimulation by external signals, such as IGF-1 or EGF, DG contributes to the activation of protein kinase C (PKC) [16–17]. This can then lead to cellular effects involving cell proliferation and differentiation [18]. More recently, studies show that DG may also modulate Rho and Ras proteins [19–20]. Kris et al. have shown an increase in epidermal DG following calorie restriction suggesting a connection between DG levels and energy balance [21].

Previous analyses of plasma or tissue TG or DG have been done using total quantity measurements within cells or fatty acid composition as opposed to investigating individual TG and DG species. The recent developments of tandem mass spectrometry combined with electrospray ionization have made the global analysis and quantification of TG and DG profiles possible [22–24]. Here, the impact of weight control on TG and DG lipid profiles in the plasma and skin tissue of TPA-treated mice is described. We found total plasma TG species were significantly decreased following a pair-fed diet (animals fed a similar amount of food as sedentary control animals) combined with exercise as well as animals receiving 20% less total calories from carbohydrates and fat (DCR). Both plasma and tissue TG species containing fatty acid chain lengths of 18:1 (number of carbons: number double bonds) were significantly decreased following DCR. In regards to DG, the most significant changes occurred in the plasma. DG species containing fatty acids with lengths 16:1 and 18:1 were significantly decreased following pair-feeding combined with exercise and DCR. Considering the significant role of TG in energy storage and DG in cellular signaling our findings surrounding the effects of weight control on individual TG and DG species in plasma and skin tissue following TPA exposure may provide additional insights into early-stage cellular mechanisms of weight control in cancer prevention.

## Materials and Methods

### Animals and Treatments

This study was carried out in strict accordance with the recommendations in the Guide for the Care and Use of Laboratory Animals of the National Institutes of Health. The protocol was approved by the Institutional Animal Care and Use Committee (IACUC) for Kansas State University (Permit Number: 3019). Female CD-1 mice at 8 weeks of age were purchased from the Charles River (Roanoke, Illinois). Mice were randomly divided into four groups: ad libitum feeding and sedentary (control, n = 10), ad libitum feeding with exercise (AL+Exe, n = 12), pair feeding with exercise (PF+Exe, n = 13) and dietary calorie restriction (DCR, n = 10). Ad libitum feeding groups (control and AL+Exe) were allowed to freely obtain the basal diet (AIN-93M) while the pair-fed exercise group (PF+Exe) was fed daily the same amount as the sedentary control mice. The time of daily feeding for PF+Exe and DCR groups was afternoon. The DCR group was sedentary and was fed a diet of 20% DCR, compared to control, for 8 weeks. Diets for all groups were custom made and provided by Harlan Teklad (Madison, WI). The DCR diet contained 20% less total calories from carbohydrates and fat when compared to the basal AIN-93M diet, and contained an increased amount of protein and micronutrients to maintain the same level as the basal diet. The amount of food given to DCR and PF+Exe group was calculated based on the previous week’s food consumption by control mice. Water was provided ad libitum to all mice. A speed adjustable rodent treadmill (Boston Gears, Boston, MA) was used for mice exercise. To take into account the biological clocks of nocturnal rodents, the light cycle was adjusted for mice to run nighttime exercise. After 2 weeks, the exercise groups ran on the treadmill at 0% incline, 13.4 m/min, 60 min/day and 5 days a week for 10 weeks. The mice were fed until the last day, but exercise was stopped 24 hours before the mice were sacrificed. All mice were housed individually at 24 ± 1°C and 60% relative humidity with 12 hr light / 12 hr dark cycle. Body weight and food consumption were recorded every week.

At the end of the experiment, the dorsal skin of each mouse was shaved and treated with 6.4 nmol 12-O-tetradecanoylphorbol-13-acetate (TPA) in 200 μL acetone. Mice were euthanized by CO_2_ two hours after TPA treatment. Blood was collected in heparin-coated tubes via a heart needle-stick and plasma samples were isolated by centrifugation at 1,000 x g for 10 minutes at 4°C. The dorsal skin samples were snap-frozen in liquid nitrogen immediately. Both plasma samples and skin tissues were kept at -70°C until further lipid extraction.

### Lipid extraction

For plasma samples, methanol (1.5 μl) was added to a 200 μl plasma sample, and the tube was placed on a vortex mixer. For skin tissues, 0.4 g of skin tissue were combined with liquid nitrogen and ground into a powder. Then two volumes of solvent [chloroform: methanol (1:2; v/v) with 0.01% butylated hydroxytoluene (BHT)] were added to 1 part plasma sample (v/v) or skin tissue (v/w) and shaken well. To the mixture, 1.25 volumes each of chloroform and water were added, the mixture was shaken and subjected to centrifugation at 200 x g. The lipid-containing lower layer was collected. Addition of chloroform and water, shaking, and centrifugation was repeated two additional times. The combined lower layers were washed with 300 μl 1.0 M KCl, then with 300 μl water and transferred to a new tube. Each plasma or skin lipid extract was dried under a stream of nitrogen, dissolved in 1000 μL of chloroform, transferred to a 2-mL pre-cleaned glass vial, and stored at -70°C until lipidomics analysis.

### Analysis of Neutral Lipids by Lipidomics

5 μl of lipid extract and an appropriate internal standard (4.7 nmol of di-15:0 DG or 3.1 nmol of tri-17:1 TG) were combined with chloroform-methanol-300 mM ammonium acetate in water (300:665:35; v/v/v) in a final volume of 1 ml. For plasma, the mixture was subjected to centrifugation for 15 minutes to pellet particulates. These unfractionated lipid extracts were introduced by continuous infusion into the electrospray ionization (ESI) source on a triple quadrupole mass spectrometry (MS) system (API 4000; Applied Biosystems/MDS Sciex, Ontario, Canada). Samples were introduced using an autosampler (LC Mini PAL; CTC Analytics AG, Zwingen, Switzerland) fitted with the required injection loop for the acquisition time and presented to the ESI needle at 30 μl/min.

The analysis of the samples by mass spectrometry was performed at the Kansas Lipidomics Research Center (Division of Biology, Kansas State University), using a modification of methods previously described [22–24]. Ammoniated adducts of DG and TG undergo characteristic loss of a single neutral species, as RCOOH+NH3, for each fatty acyl chain present in the molecules. DG and TG molecules were detected by multiple neutral loss (NL) scans. The monitored neutral losses were selected according to the known composition of fatty acids found in mouse lipids. The following neutral losses were scanned in detecting DG and TG molecules: 14:0 (NL 245), 15:0 (NL 259, internal standard), 16:0 (NL 273), 16:1 (NL 271), 18:0 (NL 301), 18:1 (NL 299), 18:2 (NL 297), 18:3 (NL 295), 20:3 (NL 323), and 20:4 (NL 321).

Instrumental parameters were: collision gas pressure at 2 (arbitrary units); collision energy using nitrogen in the collision cell, 20 V; declustering potential, 100 V; entrance potential, 14 V; source temperature (heated nebulizer), 100°C; interface heater was on; +5.5 kV or -4.5 kV was applied to the electrospray capillary; curtain gas set at 20 (arbitrary units); and the two ion source gases were set at 45 (arbitrary units). Each neutral loss spectrum was scanned from *m/z* 500 to 950 over 4 s, and the scan was repeated 30 (TG) or 37 (DG) times (2.0–2.5 min total time per NL scan).

The background of each spectrum was subtracted, the data were smoothed, and the peak areas were integrated using a custom script and Applied Biosystems Analyst software. Isotopic overlap corrections were applied, and the DG and TG MS intensities were identified and quantified in comparison to the internal standards. Data are presented as normalized MS intensity per μl plasma or per mg of tissue. A normalized MS intensity equal to 1 means that the lipid of interest had the same MS intensity as 1 nmol of the corresponding internal standard.

### Statistical Analysis

One-way ANOVA was used to analyze the body weight. Principal component analysis was used to analyze the similarity of the four treatment groups. Total plasma and tissue concentrations of DG and TG: One-way ANOVA was performed followed by Dunnett’s test to compare significance between treatment groups. Individual TG and DG species for plasma and tissue samples: Two-way ANOVA was performed followed by Tukey’s multiple comparisons test. Minimum level of significance was set at p<0.05. Data is presented as mean ± SEM. Statistical analyses were performed using GraphPad Prism 6.01.

## Results

### Impact of Weight Control via Calorie Restriction and/or Exercise on Body Weight

**Fig 1:**
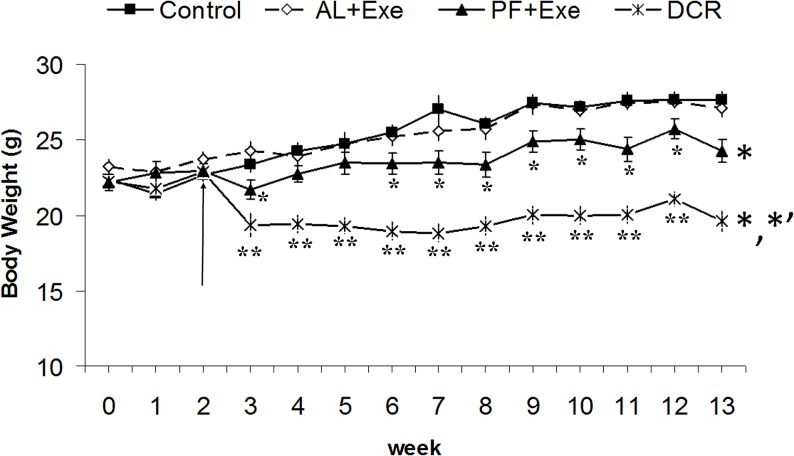
CD-1 mice body weights following various treatment groups. The groups include: control, al libitum feeding with exercise (AL+Exe), pair feeding with exercise (PF+Exe) and dietary calorie restriction (DCR). Animals were adapted to surroundings and underwent treadmill training during the first two weeks. The arrow indicates the beginning of dietary calorie restriction and physical exercise. Results presented as mean ± SE, n = 10–13 animals/group. *p<0.05 (compared to control), **p<0.01 (compared to control). For side asterisks: * (compared to control group), *’ (compared to PF+Exe).

The body weight of all experimental animals can be seen in Fig. 1. Sedentary control CD-1 mice had a weight increase of 24.3% over the course of the study (starting weight 22.2 g, end weight 27.6 g). There was no significant difference in body weights between AL+Exe and sedentary controls. In contrast, PF+Exe animals had a significantly lower body weight at the end of the study when compared to sedentary control animals. Animals in the DCR group had the most dramatic results with their weight being significantly decreased compared to both sedentary control and PF+Exe animals.

### Principal Component Analysis of the TG and DG Species of Plasma and Skin

**Fig 2:**
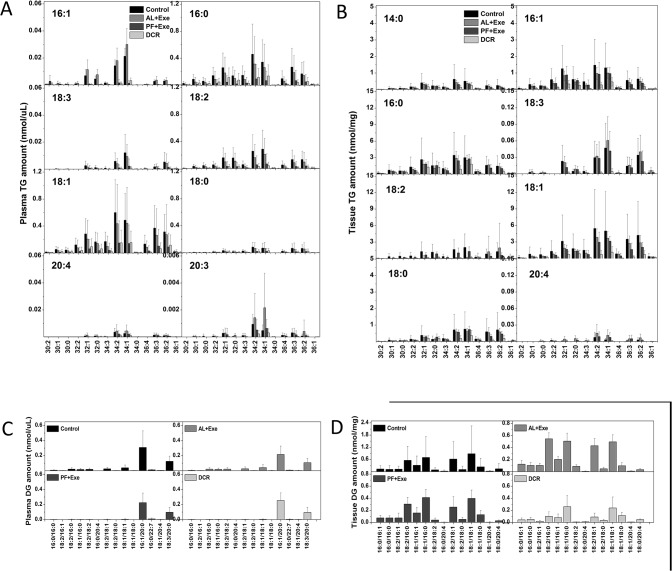
TG and DG molecular species following various treatments. A) Plasma TG. B) Tissue TG. C) Plasma DG. D) Tissue DG. For TG (Fig. 2A and 2B), one fatty acid is indicated in each panel, and the other two are combined and shown in the horizontal axis. Data presented are mean± SE. n = 10–13 animals/group.

Fig. 2 shows the TG and DG molecular species that were analyzed in the plasma samples (Fig. 2A and 2C) and tissue samples (Fig. 2B and 2D) of each treatment group. Within plasma samples, TG containing fatty acids with 16:0 and 18:1 with corresponding lipid pairs totaling 34:2 and 34:1 were most abundant. Overall, higher concentrations of TG were found within tissue samples as compared to plasma samples. Similar to plasma TG, fatty acids containing 16:0 or 18:1 with corresponding lipid pairs totaling 34:2 and 34:1 were most abundant. For plasma DG, all treatment groups behaved similarly. The 16:1/20:0 DG was most abundant in all treatment groups (Fig. 2C). Lastly, when tissue samples were obtained from each treatment group and analyzed for DG, there were no major changes in lipid profiles across groups (Fig. 2D).

**Fig 3:**
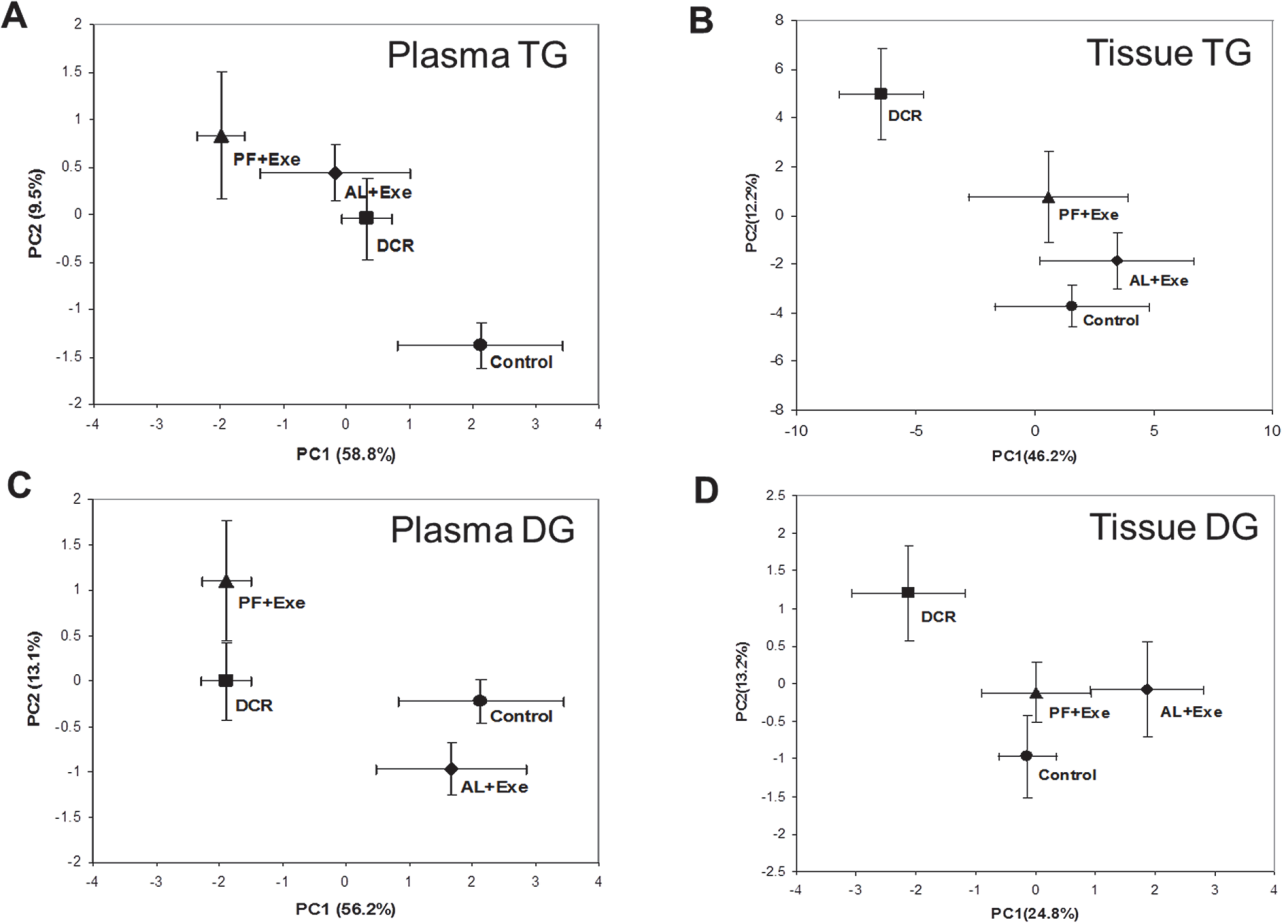
Principal component analysis for all treatment groups. A) Plasma TG. B) Tissue TG. C) Plasma DG. D) Tissue DG. Data presented are mean± SE. n = 10–13 animals/group.

Based on data presented in Fig. 2, principal component analysis was used to visualize the lipid profiles between the four treatment groups. Fig. 3 shows the biplot for plasma TG (A), tissue TG (B), plasma DG (C) and tissue DG (D). The results for plasma TG show a correlation between PF+Exe, AL+Exe and DCR treatment groups, all of which are separate from the sedentary control group (Fig. 3A). When lipid data from tissue TG was plotted, DCR animals were separate from the other treatment groups (Control, AL+ Exe and PF+Exe) which were clustered together (Fig. 3B). Data from plasma DG was different in that there was no major clustering of treatment groups (Fig. 3C). Lastly, similar to the biplot obtained from tissue TG, DCR animals were separate from the other treatments group that were clustered together (Fig. 3D).

### Impact of Weight Control via Calorie Restriction and/or Exercise on Plasma and Tissue TG in CD-1 Mice

**Fig 4:**
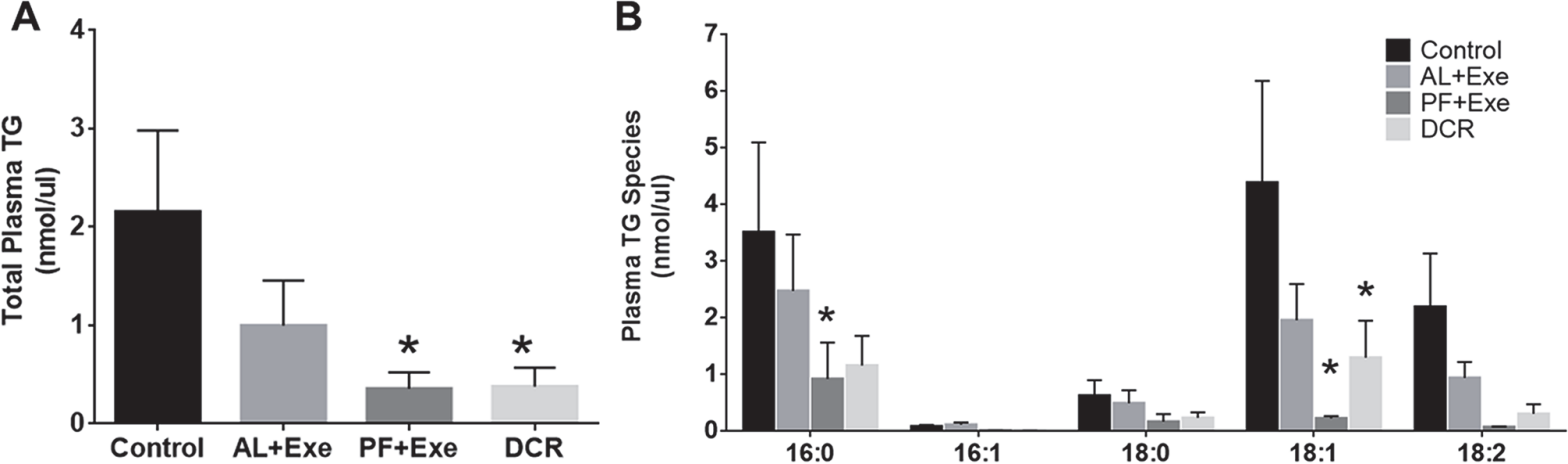
Plasma TG (normalized MS intensity per μl) following various treatments. A) Total plasma TG from all treatment groups. B) Plasma TG obtained using fatty acyl scans for all treatments. Presented species contain at least one of the indicated fatty acids. The fatty acid presented in the figure were the most abundant. Normalization is to the intensity of the TG internal standard. Data presented are mean± SE, n = 10–13 animals/group. *p<0.05 as compared to control.

**Fig 5:**
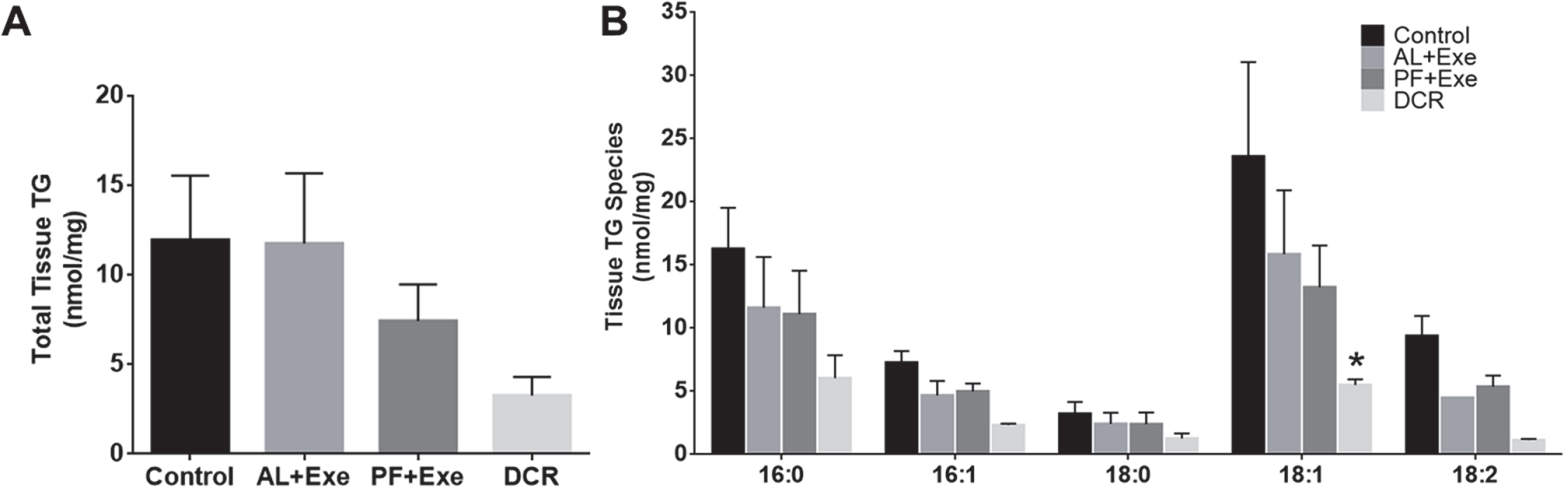
Tissue TG (normalized MS intensity per mg) following various treatments. A) Total skin tissue TG from all treatment groups. B) Individual skin tissue TG obtained using by fatty acyl scans. Presented species contain at least one of the indicated fatty acids. The fatty acids presented in the figure were the most abundant within samples. Normalization is to the intensity of the TG internal standard. Data presented are mean± SE, n = 10–13 animals/group. *p<0.05 as compared to control.

Fig. 4A contains data representing normalized total plasma TG levels in all treatment groups. Overall, there was a significant decrease in total plasma TG in PF+Exe (p = 0.019) and DCR (p = 0.020) groups compared to sedentary control animals (Fig. 4A). When viewing the most abundant fatty acids within plasma TG samples, there was a consistent decrease in TG concentrations following PF+Exe and DCR treatments compared to control animals. Significant changes were seen within PF+Exe and DCR groups in TG containing fatty acid lengths of 16:0 (PF+Exe, p = 0.023) and 18:1 (PF+Exe, p<0.0001; DCR, p = 0.008) when compared to TG of the same length in the sedentary control group (Fig. 4B). While normalized total tissue TG had a similar trend to total plasma TG of decreasing following PF+Exe and DCR treatment, there were no significant differences between treatment groups (DCR compared to Control p = 0.12) (Fig. 5A). When data was graphed for the abundant fatty acids in tissue TG, the most dramatic changes were seen in TG containing fatty acid chains 18:1 (Fig. 5B). There was a significant decrease in TG containing fatty acids with length 18:1 in DCR treated animals (p = 0.0009) when compared to control animals with TG containing fatty acids with length 18:1.

### Impact of Weight Control via Calorie Restriction and/or Exercise on Plasma and Tissue DG in CD-1 Mice

**Fig 6:**
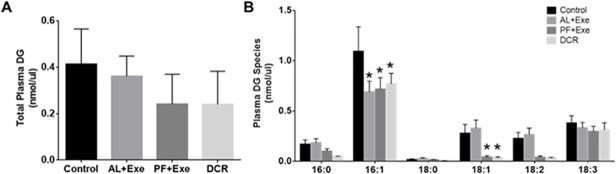
Plasma DG (normalized MS intensity per μl) following various treatments. A) Total plasma DG from all treatment groups. B) Individual plasma DG acyl group for all treatments. Presented species contain at least one of the listed fatty acids. The fatty acids presented in the figure were the most abundant within samples. Normalization is to the intensity of the DG internal standard. Data presented as mean± SE, n = 10–13 animals/group. *p<0.05 as compared to control.

**Fig 7:**
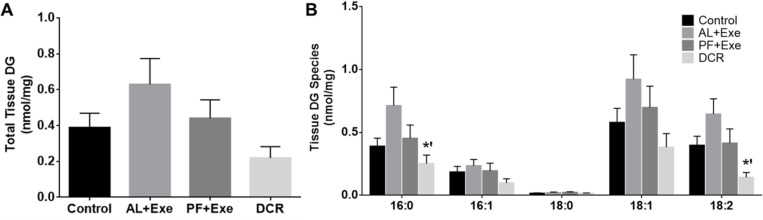
Tissue DG (normalized MS intensity per mg) following various treatments. A) Total skin DG from all treatment groups. B) Individual skin DG acyl groups for all treatments. Presented species contain at least one of the listed fatty acids. The fatty acids presented in the figure were the most abundant within samples. Normalization is to the intensity of the DG internal standard. Data presented as mean± SE, n = 10–13 animals/group. *’p<0.05 as compared to AL+Exe.

The effect of weight control on normalized total plasma DG species is shown in Fig. 6A. Overall, none of the treatment groups had a significant effect on total plasma DG levels when compared to the control group or when compared to each other (Fig. 6A). When individual plasma DG species were analyzed, there was a significant decrease in species containing fatty acid lengths of 16:1 in all treatment groups (AL+Exe, p = 0.0007; PF+Ex, p = 0.0016; and DCR, p = 0.015) when compared to the sedentary control group (Fig. 6B). Additionally, there was a significant decrease in DG species with fatty acids of length 18:1 in PF+Exe (p = 0.019) and DCR (p = 0.014) groups as compared to control animals (Fig. 6B). Similar to total plasma DG, normalized total tissue DG found within all treatment groups were not significantly different than the control group or when compared to each other (Fig. 7A). When individual tissue DG species were investigated, only the DCR treatment groups showed significant decreases in DG species containing fatty acids lengths of 16:0 (p = 0.008) and 18:2 (p = 0.003) (Fig. 7B). It should also be noted that these significant levels occurred when the groups were compared to the AL+Exe treatment group.

## Discussion

This study found that weight control via exercise with paired feeding or dietary calorie restriction decreased body weight and resulted in a significant impact on TG and DG profiles in both plasma and skin tissue of CD-1 mice. Exercise with pair feeding (PF+Exe) or 20% dietary calorie restriction (DCR) significantly decreased body weight compared to sedentary control mice, while ad libitum feeding resulted in an increase in body weight over the course of the study (Fig. 1). The body weight increase in AL+Exe animals was not significant compared to the weight gain in sedentary control animals. These results are consistent with our previous findings and others in APC^Min^ mice and SKH mice [7, 25–26]. The treadmill exercise, 13.4 meters /min for 60 minutes a day, is considered moderate intensity. As such, it is possible that mice within the ad libitum group had an increased calorie intake. Thus, exercise without consideration of dietary intake may not produce a sufficient negative energy balance and therefore not result in significant loss of body weight. When the food consumption of exercised mice was adjusted to that of sedentary control (PF+Exe), the body weight was significantly decreased (Fig. 1). The data suggests that to maintain body weight through exercise, dietary intake should be considered as an important factor.

In regards to TG species found within the plasma of our experimental animal groups, we observed significant decreases in lipid species following PF+Exe and DCR treatments (Fig. 2A and Fig. 4). The TG lowering effect by exercise and DCR has previously been shown by Cullinane et al. Their experiments showed that following one hour high intensity exercise total plasma TG was reduced by 22% in humans [12]. Subsequently, it was suggested that the exercise-induced lowering of plasma TG was dependent on energy expenditure [27], and only negative energy balance was effective in decreasing total plasma TG [28]. Our current data for individual TG species confirms these results as plasma TG species containing a fatty acid of length 16:0 or 18:1 were most affected by PF+Exe and DCR treatments. The presented results may also help explain our previous findings that PF+Exe and DCR, but not AL+Exe, significantly decreased the percentage of fat as analyzed by dual-energy x-ray absorptiometry scan [7].

In regards to plasma DG species, lipids containing fatty acids of length 16:1 and 18:1 were most affected following PF+Exe and DCR treatments (Fig. 6B). DG, a known second messenger, may bind and activate proteins such as PKC thereby leading to cell growth and proliferation [18]. It was found that different DG species may have different affinities when binding to PKC isomers [29–30]. Shinomua et al., found that saturated and trans-unsaturated free fatty acids cannot bind and activate cPKC, but cis-unsaturated fatty acids such as oleic (18:1 ω9), linoleic (18:2 ω6) and linolenic (18:3 ω3) can enhance cPKC phosphorylation and activation [30]. Studies have shown that DG with polyunsaturated fatty acyl are more active in PKC binding, while saturated DG appear to be poor PKC activators [31–33]. In SENCAR mice skin, PKCα and PKCζ protein expression were significantly reduced by calorie restriction [34]. While we did not specifically measure PKC in our samples, there were noticeable decreases (although not significant) in polyunsaturated fatty acyl groups (PUFA) following calorie restriction or pair-feeding and exercise in plasma DG (Fig. 6B) and a significant effect of caloric restriction on PUFA in tissue samples (Fig. 7B). These results suggest there could be reduced PKC signaling within our model.

We are aware of some discrepancies between our data and previously published literature. Kris et al. found that calorie restriction increased total DG in the skin of SENCAR mice [21]. Our study, however, showed no change in total skin DG in calorie restricted mice and exercised and pair-fed mice when compared to controls (Fig. 7A). This may be due to the use of different DCR protocols. While our DCR protocol used 20% reduction in energy (i.e. fats and carbohydrates), Kris et al. used a protocol that resulted in 40% restriction of energy [21]. Differences in energy consumption protocols, in regards to DCR, have been shown to result in experimental variations [4]. Additionally, the difference in mouse strain could have had an effect on lipid analysis. Therefore, while our data provides the first step in identifying individual DG species, subsequent studies will have to be conducted to obtain a clearer picture of which DG species are most important following weight control.

Studies investigating the effects of calorie restriction on longevity/aging have reported changes to membrane composition that include decreases in long chain PUFA and decreases in the degree of unsaturation of membranes [35–36]. These changes are linked to resistance of membranes to lipid peroxidation and can also have an effect on membrane structure [37–38]. Interestingly enough, our presented data shows decreases in plasma and tissue PUFA following DCR or PF+Exe (Fig. 2) but in the context of early-stage carcinogenesis. Using previous studies as a guide, follow-up studies of the presented data could include investigation of lipid peroxidation or a thorough analysis of membrane structure using the current animal model and treatment groups. Overall, the data presented in this study contributes to the current literature regarding the effects of weight control (either by exercise or DCR) on specific TG and DG lipid species. Further studies to analyze the changes of these specific lipid species will aim to provide insight into the beneficial effects of weight control on cancer prevention.
